# Is the Cis-Element CACCC-Box a Master Regulatory Element during Cardiovascular Disease? A Bioinformatics Approach from the Perspective of the Krüppel-like Family of Transcription Factors

**DOI:** 10.3390/life14040493

**Published:** 2024-04-11

**Authors:** Juan Andrés García-Loredo, Michelle G. Santoyo-Suarez, Oscar Rodríguez-Nuñez, Diego Francisco Benitez Chao, Elsa N. Garza-Treviño, Patricio Adrián Zapata-Morin, Gerardo R. Padilla-Rivas, Jose Francisco Islas

**Affiliations:** 1 Departamento de Bioquímica y Medicina Molecular, Facultad de Medicina, Universidad Autónoma de Nuevo León, Monterrey 64460, Nuevo León, Mexico; andres.garcialrd@uanl.edu.mx (J.A.G.-L.); s.antoyo@hotmail.es (M.G.S.-S.); oscar.rodrigueznu@uanl.edu.mx (O.R.-N.); diego.benitezch@uanl.edu.mx (D.F.B.C.); elsa.garzatr@uanl.edu.mx (E.N.G.-T.); gerardo.padillarv@uanl.edu.mx (G.R.P.-R.); 2 Laboratorio de Micología y Fitopatología, Facultad de Ciencias Biológicas, Universidad Autónoma de Nuevo León, San Nicolás de los Garza 66451, Nuevo León, Mexico; patricio.zapatamor@uanl.edu.mx

**Keywords:** Krüppel-like factor, transcription factors, CVDs, CACCC-box, diabetes, SP1

## Abstract

The CACCC-box motif emerges as a pivotal cis-regulatory element implicated in diverse developmental processes and diseases, particularly cardiovascular diseases (CVDs). This study centers on the intricate interplay between the CACCC-box and its binding proteins such as: the Krüppel-Like Family (KLF) of transcription factors as primary effectors in the context of CVDs. Our analysis was through a bioinformatics approach, which revealed significant transcriptional activity among KLF subgroup 2, exhibiting the highest number of interactions focusing on the established roles: pluripotency, cancer, and cardiovascular development and diseases. Our analysis reveals KLF’s interactions with GATA4, MEF2C, NKX2.5 and other ~90 potential genes that participate in the regulation of the hypertrophic environment (or CVDs’ Environment). Also, the GO analysis showed that genes containing the motif CACCC were enriched for multiple CVDs; in combination with STRING analysis, these results pointed to a link between KLFs and these diseases. The analysis further identifies other potential CACCC-box binding factors, such as SP family members, WT1, VEZF1, and -SALL4, which are implicated in cardiac contraction, remodeling, and inflammation processes.

## 1. Introduction

The gene is the most basic unit of information; contained within itself are the blueprints required for the development of all the final bioactive products in the cell, whether it may be a miRNA or other small RNA, all the way to a messenger RNA leading to a protein. In addition to these products, there is further information pertaining to the gene’s own expression. Importantly, regulation is an integral part of expression, a process controlled through elements found in the gene, particularly sequences in the promoter region [1,2].

Gene expression is a process regulated by transcription factors in which there is activation or repression of the transcription activity through their binding to specific DNA motifs mediated by their DNA-binding domains [3]. DNA motifs can be seen as short conserved sequences (ranging from 2 to 20 bp) to which sets of transcription factors (or entire families) bind. One such example of a DNA motif is CACCC-box, which serves as a binding site for several transcription factors, including the Krüppel-Like Factor (KLF) family [4], the Specificity Protein (SP) family [5], the Wilms tumor gene (WT1) [6], Vascular Endothelial Zinc Finger 1 (VEZF1) [7], and Splat-Like Transcription Factor 4 (SALL4) [8].

As a regulatory element, the DNA-motif CACCC-box, alongside other cis-elements such as TATA and CAAT boxes, work together in promoters, such as seen in the cardiac B-type Natriuretic Peptide (BNP) gene, wherein deletions result in reduced transcriptional activity in postnatal cardiomyocytes, an activity regulated by KLF-13 [9,10,11,12].

Out of the earlier-mentioned transcription factor families which bind to the CACCC-box, much research has been devoted to the KLFs for their roles in diverse heart-related processes such as the maturation of cardiac myocytes, and their dysregulation is associated with several CVDs, as exemplified by cardiomyopathies, infarctions, left ventricular hypertrophy, and diabetic cardiomyopathies [4,13,14,15,16]. Particularly, CVDs continue as the leading cause of death worldwide. According to a recent publication by the American College of Cardiology “CVDs are a persistent challenge that led to an enormous number of premature and preventable deaths”; specifically, ischemic heart disease ranks #1 in mortality, leading to 108 deaths per 100,000, and overall, CVD-related deaths accounted for 19.8 million in 2022 [17]. As stated, KLFs have been shown to play a crucial role in the progression and control of CVDs [4]. Some examples of regulating CVDs by KLFs can be seen in atherosclerosis, as the expression of KLF-5 switches a proliferative phenotype in vascular smooth muscle cells, while the repression of KLF-2 leads to an inflammatory state and vasculature remodeling [18,19,20]. During myocardial infarction, there is a noted elevation of KLF-4, which induces the secretion of collagen type I and III via the TGF-β1/Smad3 pathway, contributing to myofibroblast differentiation [21,22,23]. In diabetic cardiomyopathy, KLF-5 increase has been shown to upregulate NADPH oxidase 4, a primary cause of cardiomyocyte superoxide accumulation; moreover, KLF-5 further leads to the activation of the serine palmitoyl transferase [SPT] long-chain base subunits 1 and 2, enzymes involved in ceramide synthesis. This process, in turn, changes the lipid landscape of the heart, further contributing to the cardiomyopathy phenotype [13,24]. Interestingly, within a diabetic patient’s mutations in KLF-15, such as rs9838915, they are associated with an increase in the risk of heart failure, as rs9838915 is linked to increased LV mass and thickening of the septal wall [25]. KLF-13 loss-of-function variants in the heart have been associated with double-outlet right ventricle and ventricular septal defects [12], hence reducing the activity of GATA-6, GATA-4, and ANP promoters [12]. Additional KLF-13 mutations have also been connected to congenital heart defects; particularly, KLF-13 is an activator of VEGF-a and ANP. Nonetheless, its mutants are linked to the bicuspid aortic valve, patent ductus arteriosus, and ventricular septal defect [10]. Finally, in stroke there is a reduction in the expression of KLF-2 which directs proinflammatory effects by permitting the expression of the NF-B/p65 pathway [26].

Other examples of regulatory effects structured by the CACCC-box can be seen by the governing effects of the SP family [27]. Among SP members, the transcriptional factor Sp1 has been reported to work with KLF-4 and even overlap its binding site. Particularly, during esophagus carcinoma, levels of Sp1 rise, disrupting KRT19 regulation by KLF-4, which leads to malignant transformation and metastasis [27].

As mentioned, gene expression is a tightly controlled process facilitated in part by the accessibility of the DNA and regulated by the elements to which transcription factors can bind. Therefore, we sought to take a bioinformatic approach and investigate gene enrichment, especially targeting diseases and metabolic pathways by establishing associations between genes containing the CACCC-box upstream from their promoter. Furthermore, we investigated their relation to the KLF family as regulators or key players in CVDs to comprehensively understand the interplay between these genetic elements.

## 2. Materials and Methods

### 2.1. Raw data Collection and Web Scraping

The genome annotation of *H. sapiens* GRCh38/hg38 (2013) from the Ensembl project series was utilized in this study, employing the Signal Search Analysis Server (SSAS) software package (https://epd.expasy.org/ssa/findm.php, accessed on 23 April 2023) with the promoter prediction tool. The -CACCC- motif sought 150 base pairs upstream and 300 bp downstream of the TATA-Box/Goldberg–Hogness Box functional site, selecting the best options.

The sequences containing the TATA-Box motif were obtained, and an algorithm was used to search for the -CACCC- motif within the first 150 base pairs upstream and 300 bp downstream. Subsequently, these sequences underwent multiple sequence alignment analyses using BLAST. Given the large number of sequences, an algorithm utilizing Web Scraping and Python 3.9.13 code with the Selenium WebDriver 4.8.0 was employed to extract the gene names associated with each sequence.

With the Selenium Python library and the web driver functionality, the program automates the process of inputting the search query on the BLAST NCBI website. The web driver interacts with the website’s interactive slot, allowing the script to enter the desired query and initiate the search engine. Once the search is performed, the web scraper identifies the specific result page containing the relevant information. The required data from the result page are extracted by analyzing the website’s HTML structure and utilizing techniques such as HTML parsing and text pattern matching.

The extracted data were transformed into a CSV, for further analysis and storage, to be easily processed and utilized for further bioinformatic analysis.

### 2.2. Implementation with Python and Selenium

The Python programming language and the Selenium library (v. 4.9.0) were used to automate web scraping on the BLAST NCBI website. By leveraging the Selenium web driver and the Python framework, a web bot was created to complete the search form and crawl through the links on the main page. Specific parameters were established to narrow down the search to *Homo sapiens* (taxid:9606) and (RefSeqGene[Title]) OR gene[Title] in the Entrez query. The Python code utilizes XPath and EC.presence_of_element_located condition to target and extract the desired elements, ensuring that only relevant data are included. The extracted gene information can be printed for further processing.

### 2.3. MEME

A randomized sampling of 40 sequences was conducted using Python from the dataset containing 3044 sequences to observe the filtered motif. The most representative motifs were selected by XSTREME (5.5.5), a motif discovery and enrichment analysis with *p*-value threshold of 0.05 and motif sequences in the range of 8 to 15 nucleotides. Similar motifs to those searched were compared within the same platform using Tomtom (5.5.5), a motif comparison tool.

### 2.4. STRING

The genes were categorized into two groups, direct and indirect, employing STRING (11.5) as TSV format and visualizing the interactions using Excel version 2403 The direct group comprised genes exhibiting various levels of interaction, ranging from weak to strong, with members of the KLF family. Conversely, the remaining genes were assigned to the indirect group.

### 2.5. ShinyGO

We employed ShinyGO (version 0.77), a specialized analysis tool tailored to the target species, *Homo sapiens*, to analyze the gene sets. We set a statistical significance threshold of 0.05 (false discovery rate, FDR), ensuring robust results.

To focus on relevant findings, we applied filtering criteria by considering only pathways with a minimum of 10 genes. This step aimed to reduce noise and present meaningful results. Ultimately, the analysis yielded a total of 30 metabolic pathways that were statistically significant and enriched with genes related to KLFs.

The pathways analysis provided valuable insights into the biological processes, cellular components, molecular functions, and diseases associated with the gene sets. This approach facilitated the interpretation and analysis of the results, contributing to a comprehensive understanding of the interaction and functional implications of genes with KLF transcription factors.

Using the same dataset, relevant information regarding cardiovascular diseases could also be extracted through the representative KEGG pathway maps.

### 2.6. Enrichr and Appyter

Continuing from the previous point, utilizing the same dataset, we conducted a similar analysis using Enrichr. Specifically, we focused on disease analysis, utilizing the JENSEN database as a resource. Coupled with this analysis, the Appyter platform provided various visualization options for the results. This approach allowed us to identify the genes associated with the presented cardiovascular diseases and facilitated the creation of corresponding tables for further exploration.

## 3. Results

**Figure 1:**
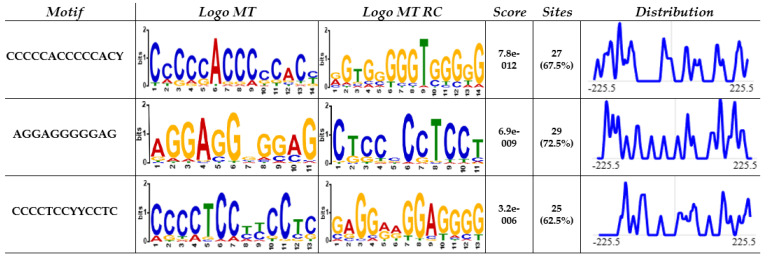
From left to right, the columns present: (1) motif sequence, (2) motif logo, (3) reverse complementary motif logo, (4) *p*-value, (5) percentage of motif occurrence in the dataset, and (6) distribution of the motive within the sequences.

**Figure 2:**
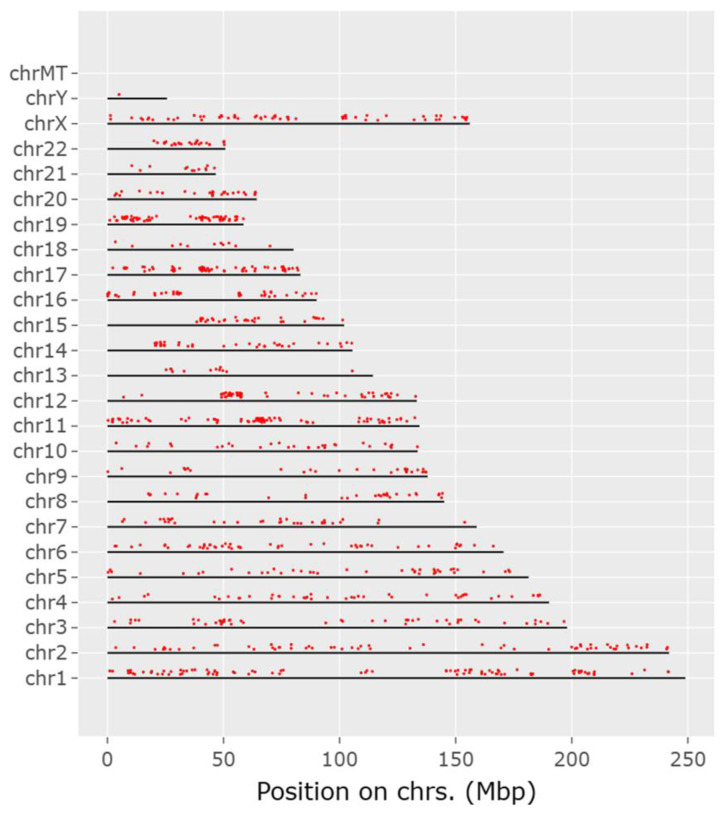
Chromosomic schematic representing CACCC-boxes (red marks) at 150 bp upstream or 300 bp downstream from known TATA-boxes.

The CACCC-box motif was initially searched using Signal Search Analysis Server (SSAS) at 150 bp upstream and 300 bp downstream of transcription start sites (TSS) 5′-TATA(A/T)A(A/T)-3′(CACCC-regions-), and Genome Reference Consortium Human Build 38 Organism: *Homo sapiens* (GRCh38) was used as reference. The results obtained a total of 3044 hits for the CACCC-box with 40 diverse matrices regions. Figure 1 shows the three highest representative core matrix regions found encompassing the core CACCC-box. CCCCCACCCCCAC(C/T) sequence was found in 67.5% of the CACCC-regions with a score value of 7.8 × 10^−12^; next, CTCCCCCTCCT was found in 72.5% of the regions and with a score value of 6.9 × 10^−9^; and finally, the sequence CCCCTCC(C/T)(C/T)CCTC was found in 62.5% of the regions and with a score of 3.2 × 10^−6^. Additional sequences can be found in Supplementary Table S1. Moreover, Figure 2 shows a representative diagram for each chromosome, denoting CACCC-box binding (red marks) for identifiable genes. Out of 3044 potential hits, these included nonsense repeats, as well as non-transcription starting points; 1174 hits were related to identifiable genes.

Our initial objective was to resolve the involvement of the KLF family; after processing, our data registered 95 hits confirmed by STRING for KLF interactions. Table 1 shows the interacting genes related to their corresponding KLF members. Surprisingly, KLF-16 did not show any gene interaction; moreover, most confirmed gene interactions are linked to the activator group (Group 2), wherein KLF-4 had the most interactions with nearly 50 hits. Opposingly, Group 1 had the least number of interactions with a total of 8 hits, mostly related to the Homeobox NK family. It is important to note that while the CACCC-box is a direct binding motif for KLFs, other important factors involved in cardiovascular regulation can potentially bind. Using the same methodology as previously mentioned, we found potential motif sites for WT1, POZ/BTB and AT Hook Containing Zinc Finger 1 (PATZ1), Lysine Methyltransferase 2A (KMT2A), Specific SP, SALL4, and VEZF1. Supplementary Figure S1 shows both the confirmed binding motif of these factors and the theoretical motifs found through our analysis. Supplementary Table S2 shows interactions between non-members of the KLF family and their target genes, including several genes which seem to be regulated either in combination KLFs and non-KLFs or in alternative modes. Some of these genes include IGF1, MEF2C, MYH11, MYOD, and TCF7L2, amongst others.

**Figure 3:**
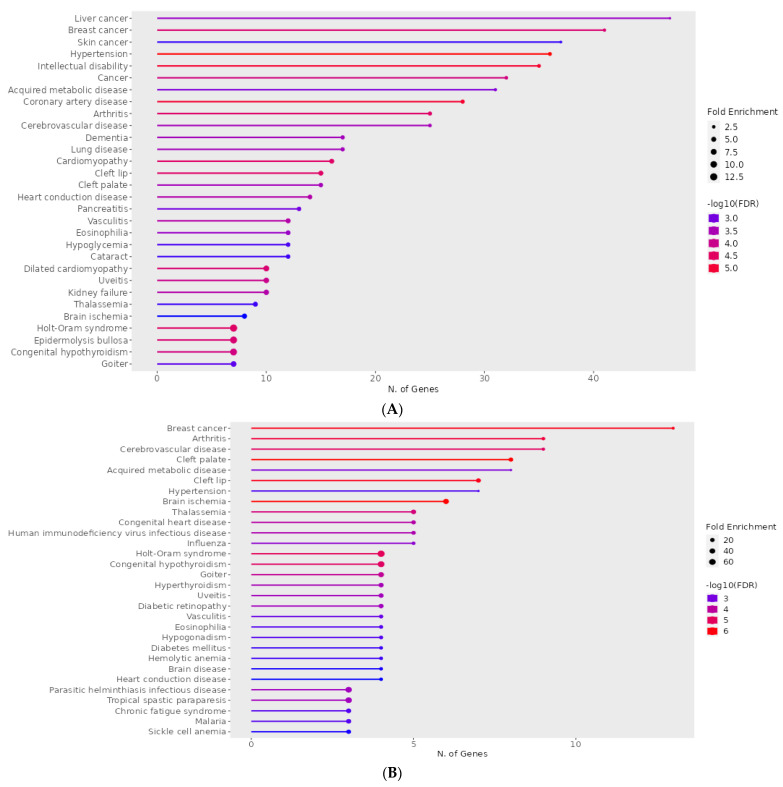
ShinyGO enrichment analysis of CACCC-box involvement in diseases: (**A**) Complete set; (**B**) KLF specific interactions. The horizontal plane correlates to the number of genes involved per disease. Fold enrichment and false discovery rate (FDR) apply to the integrated data to resolve for differentially confirmed genes indicating overrepresentation within the disease. Fold enrichment directly relates to gene target confirmation. FDR relates to the expected number of positive or significant classifications. Color lines represent FDR weight; blue represents the least statistical significance and red the highest statistical significance. Additionally, line thickness is proportional to fold enrichment.

Taking into consideration all 1174 genes, we ran a ShinyGO analysis focused on diseases using the Jessen database. Overall, there were 30 known diseases, with an overrepresentation of genes with the CACCC-motif at the TSS. Of these diseases, 4 were directly linked to cancer, and 7 to cardiovascular disease, the focus of this manuscript. Other minor representations included neurological condition, pancreatis, lung, and congenital diseases (Figure 3A). In addition, we went ahead with and ran a second ShinyGO analysis using only the 95 genes confirmed to have KLF interaction. This second analysis showed 5 similar diseases wherein CACCC-box is overrepresented (Figure 3B).

The resulting information was then taken to ENRICHR for gene identification. Table 2A shows the relation between the identified disease and the set of CACCC-box-related genes for all 1174 potential genes. Meanwhile, Table 2B denotes the relation between the identified disease and the set of CACCC-box-related genes for the 95 confirmed genes related to the KLF family. Interestingly, both tables show similar gene enrichment for Holt–Oram, hypertension, cerebrovascular disease, and heart conduction disease. Meanwhile, cardiomyopathy and coronary artery disease were only present in Table 2A, while diabetes and diabetic retinopathy were present in Table 2B. Out of all the present genes, the troponin I isoform 3 (TNNI3) was the gene most prevalent as seen in hypertension, cardiomyopathy, cerebrovascular disease, and coronary artery disease. Other highly represented genes were ICAM and IL6, both related to inflammation. Additionally, there was an important presence of the TBX family, particularly 3, 5, and 6, and troponin T isoform 2 (TNNT2).

**Figure 4:**
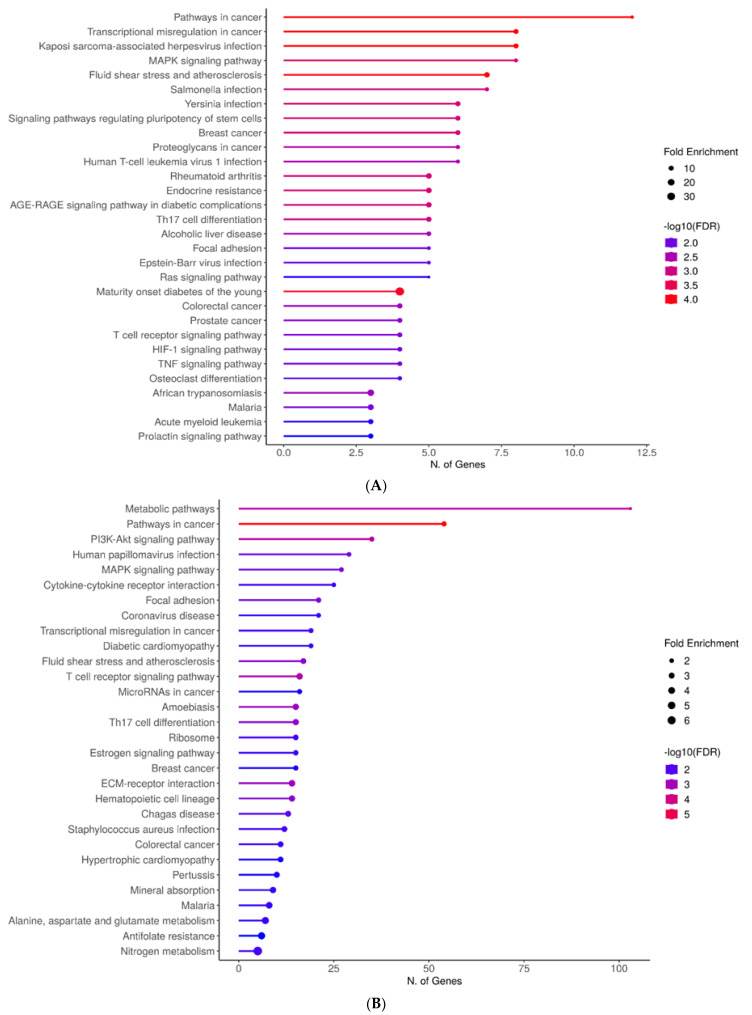
ShinyGO enrichment analysis of CACCC-box involvement in biological processes: (**A**) Complete set, (**B**) KLF-specific interactions. The horizontal plane correlates to the number of genes involved per biological process. Fold enrichment and false discovery rate (FDR) apply to the integrated data to resolve for differentially confirmed genes indicating overrepresentation within the biological process. Fold enrichment directly relates to gene target confirmation. FDR relates to the expected number of positive or significant classifications. Color lines represent FDR weight; blue represents the least statistical significance and red the highest statistical significance. Additionally, line thickness is proportional to fold enrichment.

Further ShinyGO analysis was run for the general 1174 genes which reveal their involvement in major (cardiac) metabolic pathways such as PI3K-Akt and MAPK signaling (Figure 4A); additionally, diabetic and hypertrophic cardiomyopathy-related pathways. Moreover, the 95-gene-specific analysis reveals enrichment in MAPK signaling, AGE-RAGE, RAS signaling, and HIF-1 signaling amongst other pathways (Figure 4B).

**Figure 5:**
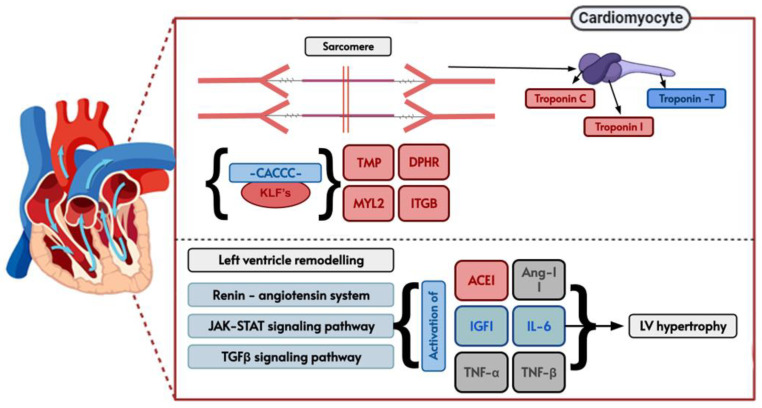
KEGG hsa05410 pathway representative of hypertrophic cardiomyopathy demonstrating biological gene interactions. CACCC-box containing genes (red) not directly related to KLF interaction (blue) with direct KLF interaction.

**Figure 6:**
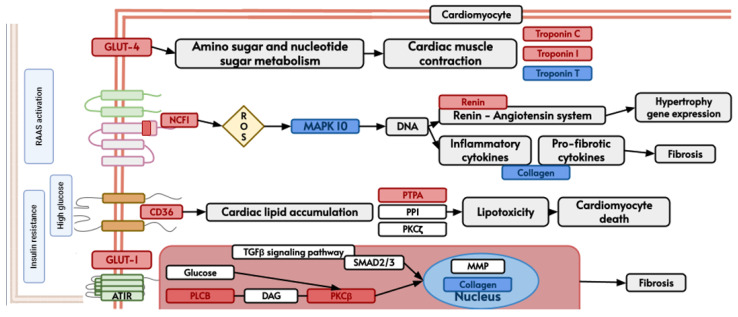
KEGG hsa05415 pathway representative of diabetic cardiomyopathy demonstrating biological gene interactions. CACCC-box containing genes (red) not directly related to KLF interaction (blue) with direct KLF interaction.

ShinyGO analysis directly links to the Kyoto Encyclopedia of Genes and Genomes (KEGG) database (https://www.genome.jp/kegg/pathway.html, accessed on 22 October 2023), to directly visualize gene interaction in the cell [28]. As a representation of cardiac disease involvement, we present in Figure 5 a schematic depiction of hypertrophic cardiomyopathy [29] and in Figure 6 of diabetic cardiomyopathy [30]. Blue-colored genes represent KLF-confirmed interacting genes, while red-colored represents the general 1174 (CACCC-motif) genes. Furthermore, to show direct protein–protein (KLF) interaction, a STRING (12.0) analysis was run for both diseases (Supplementary Figure S2). For hypertrophic cardiomyopathy, the analysis showed a direct interaction, specifically co-expression and text mining, between IL-6 and KLF-2, KLF-4, and KLF-6. Similarly, IL-6 also directly interacts with TNNT2, TNNI3, and ACE, suggesting the potential for indirect interactions mediated by IL-6, KLFs, and genes related to the sarcomere.

For diabetic hypertrophy, KLF-4 exhibits a robust interaction with COL1A1, which, in turn, demonstrates a co-expression interaction with CD36. Similarly, ACE, NCF1, REN, and SLC2A1 also display co-expression interactions with CD36. The presence of genes related to the mitochondrial cell component, such as NDUFB9, NDUFA6, NDUFA12, ATP5MC3, COX7B, NDUFA7, and ATP5P, which specifically function in mitochondrial proton transport, is also observed with a strong correlation.

## 4. Discussion

The CACCC-box motif, a well-recognized cis-regulatory element, plays a fundamental role in a diverse range of cardiac developmental processes and diseases, including CVDs. This research delves specifically into the CACCC-box and its intricate connection to CVDs, focusing on the KLF of transcription factors as the primary effectors.

The KLF family of transcription factors are zinc finger proteins consisting of 18 members and are responsible for activation and repression of transcription; particularly, several members have been linked to specific processes such as KLF-4, -5, -10, -13, -14, and -15, which relate to the cardiovascular system. In addition, KLF-4 and -7 relate to diabetes, while dysregulation of others further contributes to CVDs [4,31].

Our findings illuminate significant activity among various KLFs, particularly those of the KLF subgroup 2 recognized as transcriptional activators (Table 1). Notably, KLF-4 stands out as the KLF with the highest number of interactions, likely because of its well-established roles in pluripotency, cancer, and, most importantly, cardiovascular development and disease (Table 1 and Table 2, Figure 3, Figure 4 and Figure S2) [4,13,32,33,34]. This is further confirmed by several crucial KLF-4 interactions in our work, including those with PAX9, PAX6, TBX5, and TBX3 (Table 1 and Supplementary Figure S2). Previous research has established a link between KLF-4 and PAX9 (Table 1 and Supplementary Figure S2) during cardiovascular development, specifically regarding the crucial WNT signaling pathway. In the pharyngeal endoderm, PAX9 interacts with TBX1 and GBX2, tightly controlling the intricate process of pharyngeal arch morphogenesis [35]. Similarly, PAX6 and NKX2.2 (Table 1) have been shown to orchestrate the differentiation of neural tube ventral progenitors by mediating the sonic hedgehog signaling pathway [36].

Our exploration of the motif landscape yielded intriguing insights into pathological hypertrophy, revealing striking similarities to signaling pathways observed during early cardiac development (Figure 3). This resulted in the identification of KLF-4 interactions with genes like GATA4, MEF2C, TBX5, NKX2.5, and SRF (Table 1, Table 2 and Table S2, Supplementary Figure S2), all of which are known regulators of cardiac hypertrophy and pro-hypertrophic pathways [37,38,39]. Interestingly, KLF-15 presents a contrasting role, as it was found to repress TCF7L2 expression specifically in the postnatal heart, influencing cardiac growth. TCF7L2 is associated with diabetes and exhibits dependence on KLF-14 (Table 1 and Table S2), where reduced expression increases pre-adipocyte proliferation but impairs lipogenesis [40]. KLF-15 plays a vital role in regulating cardiac physiology, including circadian oscillations in cardiac cells and regulating genes involved in cardiac metabolism. Notably, both KLF-15 and ARNTL work in parallel to control cardiac circadian rhythms, even though no evidence of co-regulation with the ARNTL gene was observed (Table 1). KLF-15 plays a key role in binding circadian repressors REV-ERBα (NR1D1) and NCOR (Table 1), ensuring stable expression of a subset of cardiac genes [41,42].

The significant impact of hypertrophic cardiomyopathy and diabetic cardiomyopathy on cardiovascular health cannot be overstated. While hypertrophic cardiomyopathy affects approximately 1 in 500 individuals globally, with a significant proportion of cases remaining undiagnosed, diabetic cardiomyopathy emerges as a serious complication of diabetes mellitus, significantly increasing the risk of heart failure and premature death [43,44]. Both conditions highlight the critical importance of early detection, accurate diagnosis, and implementing effective management strategies to minimize the burden of cardiovascular disease on the population.

As diabetes is a condition linked to increased cardiovascular risk (Table 2 and Figure 3), it was unsurprising that our work revealed gene interaction between KLFs, particularly KLF-14, and genes associated with diabetic risk factors, such as CAMK1D, HHEX, JAZF1, and TCF7L2 (Table 1 and Table S2). Notably, TCF7L2′s ability to modulate PIK3R1 expression suggests its involvement in insulin signaling pathways, potentially mediating diabetes risk through interactions with KLF-14 [45,46].

As illustrated in Figure 4, metabolic pathways like MAP Kinases and PI3K/Akt were overrepresented, with approximately 40 genes associated with these processes, their participation, or regulation. KLF-4 emerged as the regulator of genes such as FN1, IGF1, and COL1A1 (Table 1). Upon binding to the α5β1 membrane receptor, FN1 activates the PI3K/Akt signaling pathway [35]. Additionally, the PI3K/Akt signaling pathway induces the proliferation of cardiac fibroblasts and collagen accumulation, thus contributing to fibrosis, a process associated with collagen (COL1A1) (Table 1 and Table S2) [47]. This process is a consequence of various diseases identified in the enrichment analysis (Figure 3 and Table 2), such as hypertension and its vascular remodeling process, which alters the composition of the vascular wall, leading to stiffness and loss of elasticity [48].

Furthermore, our analysis identified other factors, including members of the SP family, WT1, and others (Supplementary Table S2 and Supplementary Figure S1), that potentially bind to the CACCC-box motif. Notably, earlier studies showed that SP members 1 and 3 are further involved in contraction, as they play critical roles in calcium management by regulating the Ca^2+^ ATPase pump SERCA2 [49]. Other SP members have been shown to participate in other cardiac-related pathways, such as in the case of SP6, which can directly interact with β-catenin and modulate the expression of TCF7L2, a result confirmed by the presence of the CACCC-box in the promoter region of TCF7L2 (Table 1). These findings implicate all these members as potential regulators in electrically related CVDs.

Moreover, our results also showed regulatory effects of SP7 over COL1A1, members of the FGF and TNF families, as well as DKK-1 and IL-6 (Supplementary Table S2). This implicates SP7 in potential remodeling and inflammatory processes. Specifically, in the heart, SP7 has been previously shown to play a decremental role in favor of calcification by permitting the activity of Osteoporin and Matrix Gla protein. Downregulation of SP7 in VSMCs leads to a loss of contractile markers and paves the way for the upregulation of RUNX2 [50,51]. RUNX2 is also regulated by another CACCC-box motif binding protein KLF-5, which regulates the activity of SP7. Overall, the process of calcification further induces remodeling via inflammation, to compensate for the reduction in contractile force [51,52]. Vascular calcification, or calcium build-up in the artery wall, plays an important role in CVDs, and while in our study this process was not enriched, several key genes related to the process and other analogous processes were, such as MAPK, TGF/SMAD and HIF1 signaling, ICAM-1 activation, inflammation IL, and genes related to TNF signaling. Moreover, vascular calcification has also been widely studied in the kidney [53,54], wherein this pathological process has also been related to high levels of mortality. Studies by [54] further showed differential expression of CACCC-box motif containing genes such as TRMP7, as was seen downregulated in our study (Table 2); by contrast, DKK1, known to directly interact with KLF-4, was elevated (Table 1 and Table 2). Interestingly, both TRMP7 and DKK1 have been shown to elevate under cardiac conductivity pathologies, which lead to fibrosis and calcification [53], potentially making these genes candidate biomarkers, but further research is warranted.

Our results revealed that the CACCC-box can further serve as a complementary reverse binding site for PATZ1, known for its regulatory effect in the cell cycle, WT1, whose mutations have been associated with congenital heart defects and KMT2A, which primarily has been related with leukemia, but has been found to also associate with congenital heart defects and cardiovascular diseases by regulating gene expression essential for cardiac development and function [6,55]. In addition, other potential binding proteins found included VEZF1 and SALL4 (Supplementary Table S2). The VEZF1 gene encodes a nuclear protein highly conserved among vertebrates, regulating genes related to cardiac muscle contraction and potentially regulating dilated cardiomyopathy, suggesting that VEZF1 has a crucial role in cardiac function, and its regulation can serve as a potential therapeutic target for cardiovascular diseases characterized by impaired muscle contraction [56]. Regarding the interaction between SALL4 and KLFs in the heart, SALL4 interacts directly with members of the T-box family 3 and 5 and regulates the expression of Gap junction alpha-5 (GJA5) and KLF-4. This final regulation of KLF-4 is further seen in stem cells, as SALL4 has an important role in proliferation and survival, mediated by the Bmi-1 pathway [8]. Meanwhile, in cardiac development, another KLF, KLF-5, is involved in regulating TBX5, as KLF-5 absence reduces expression of TBX5 (Table 1, Table 2 and Table S2), contrasting with the interactions described between SALL4 and TBX5 in cardiac patterning [57]. This discrepancy highlights the complexity of regulatory networks governing cardiac development, suggesting intricate crosstalk between different transcription factors and signaling pathways.

From a clinical perspective, our study holds value at a diagnostic and potentially prognostic level. By continuing to understand the molecular mechanisms that govern gene expression, new potential biomarkers for diverse CVDs can be found and used, giving rise to predicting the development of these pathologies. It is worth noting, that through a deep understanding of the dysregulation of specific genes and pathways, targets such as downstream effectors of KLF4, 5, and 15, which are involved in hypertrophy and fibrosis, may be new avenues for treatment in conditions such as diabetic cardiomyopathy and heart failure. In addition, our finding suggested pathways like the PI3k/Akt which, when properly targeted, could attenuate the development of cardiovascular complications in diabetic patients.

Through the discovery and comprehension on interactions between KLFs and other CACCC-box binding factors, regulatory effects can be better explained. Additionally, the further exploration of adjacent motif and potential binding partners sets up future studies to have a more developed picture at a particular pathology. Targeting these interactions may offer new opportunities for therapeutic intervention, either through small-molecule inhibitors or gene editing approaches aimed at modulating the activity of specific transcription factors involved in cardiovascular gene expression.

From a diagnostic perspective, the identification of gene signatures associated with cardiovascular pathology may help identify novel biomarkers for risk stratification, early detection, and monitoring of disease progression in patients at risk of CVDs. Biomarkers derived from the transcriptional profiles of CACCC-box-regulated genes, especially those under the control of KLF family members, may offer improved sensitivity and specificity compared to existing biomarkers, enabling more accurate diagnosis and prognosis of CVDs. By leveraging the understanding of the molecular mechanisms underlying CVDs, clinicians can look into the future to tailor therapeutic interventions targeting specific pathways or gene networks dysregulated in each patient, thereby optimizing treatment efficacy and minimizing adverse effects.

## 5. Limitations

The bioinformatic approach employed in this study offers valuable perceptions into the regulatory landscape of cardiovascular gene expression. It is essential to acknowledge its inherent limitations. The stringency parameters applied in the analysis may be a risk of overlooking potentially relevant motifs or genes that do not meet the predefined criteria, which may introduce biases or inaccuracies in the results. As our study centered only on the CACCC-box motif from 150 bp upstream to 300 bp downstream of the TATA-box, hence other relevant KLF binding motifs such as GC-rich regions GC- and GT- were not explored. Therefore, several previously identified genes known to be regulated by KLFs and several processes were not enriched during this study, such as vascular wall homeostasis and atherosclerosis [58,59].

Furthermore, it is difficult to broadly evaluate regulatory mechanisms based only on transcription factors and cis-regulatory elements, especially since epigenetic regulation, including DNA methylation, histone modifications, and chromatin remodeling, influences gene expression patterns and add complexities. However, the genomic data sourced from the Ensembl project series provide widely used and reliable information. Experimental and future research efforts are necessary to further validate the functional significance of the identified genes and pathways in the context of CVDs.

## 6. Conclusions

In conclusion, this study sheds light on the intricate network of CACCC-box regulators, particularly KLFs and their interactions with various genes and pathways, predominantly in the context of cardiovascular diseases. Our findings highlight the crucial role of KLFs, especially KLF-4, in these conditions and pave the way for further exploration of their potential as therapeutic targets. Additionally, the identification of other CACCC-box binding factors like SP family members suggests their potential involvement in various CVDs, warranting further investigation. Overall, this work contributes to our understanding of the complex regulatory mechanisms underlying cardiovascular health and disease.

## Figures and Tables

**Table 1:** Krüppel-Like Family of transcription factors gene interactions according to the STRING database. The table shows direct interactions between confirmed members of the KLF family and their target genes. These direct interactions are involved in diverse processes, including cellular development and differentiation.

Family Member	KLF	Direct Interaction
1	KLF-3	*NKX2-8, NKX2-2, LLGL2*
1	KLF-8	*NKX2-8, NKX2-2*
1	KLF-12	*TFAP2A, NKX2-2, DNASE1*
2	KLF-1	*SPI1, HBZ, NKX2-8, TET2, CD44, HBB, HBE1, HBG2, GATA2, AHSP EPB42, UBC*
2	KLF-2	*GZMB, TNNT2, DUSP1, CCR7, LEF1, ZAP70, CXCR4, TEK, HAS2, CD44, FOXP3, FOS, FBXW7, ICAM1, GATA2, TRPV4, CD8A, TBX3*
2	KLF-4	*SALL4, COL1A1, SPI1, TNNT2, SOX9, MYOD1, TBX3, EED, EPAS1, EPCAM, LEF1, CDH2, NODAL, GRHL3, T, IGF1, FOS, HAS2, TBX5, TUBB3, NKX2-5, DLK1, MEF2C, GATA2, FN1, PAX9, SOX15, LEFTY2, NES, TCF7L2, FLNA, DKK1, RARG, KLF-6, KDM2B, TET2, ESR1, GFAP, CXCR4, IL6, MYH11, CD24, CD44, TCF4, PAX6, PBX1*
2	KLF-5	*SOX9, TBX3, TP63, NCOR1, FBXW7, NODAL, UCP3, GATA2, MKX, SUMO1, ESR1*
2	KLF-6	*PPP1R15A, FOS, DUSP1, KLF-4, ESR1, CYP2E1, NAT1, ANGPT1*
2	KLF-7	*PROKR2, GABPB1, TBX3, LEF1, NFIA, NKX2-8*
3	KLF-9	*NKX2-8, THRA, RB1, MAPK10, TFAP2A, ACTG2*
3	KLF-10	*NKX2-8, SMAD7, NKX2-2, FOS, FOXP3*
3	KLF-11	*HNF1A, NKX2-2, FOXP3,*
3	KLF-13	*HBB, HBE1, TBX5*
3	KLF-14	*HHEX, TCF7L2, FHL2, CAMK1D, JAZF1*
3	KLF-16	*-*
-	KLF-15	*NR3C1, NFIA, ARNTL, GATA2, MKX, NKX2-8*
-	KLF-17	*TG, NODAL, CD44, TBX3, SLC5A5*

**Table 2:** ENRICHR analysis of genes and cardiac-related diseases directly regulated by CACCC-box: (A) all genes, (B) confirmed interactions to the KLF family.

(A) Disease	q-Value	*p*-Value	Genes
Holt–Oram syndrome	2.91 × 10^−6^	0.001117367	*TFAP2B, SALL4, GJA5, TBX6, TBX5, NKX2-5, TBX3*
Hypertension	6.45 × 10^−6^	0.00148591	*ENPEP, NCF1, CUL3, NPR3, KLK4, UMOD, ADM, CACNA1D, DBH, AAAS, NR3C1, ICAM1, ADD2, GJA5, NPY, TNNI3, GGT1, DUOX2, KL, ACE, ANGPT1, CYP4F2, CAV1, ATP6AP2, MTHFR, IGF1, FOS, RHOA, SELP, IL6, TH, NEDD4, SCNN1B, CYP11B1, TRPV4, REN, ANG*
Dilated cardiomyopathy	8.98 × 10^−6^	0.001549478	*BAG3, LAMA4, TNNC1, TNNT2, ANKRD1, LDB3, TNNI3, DSG2, CRYAB, VCL*
Coronary artery disease	9.42 × 10^−6^	0.001549478	*SLC22A4, KLK4, ADM, TNFRSF11B, LDB3, AAAS, MPO, CX3CL1, THBS4, ICAM1, XIRP1, TNNI3, CD36, GGT1, LDLR, SCAI, ACE, ANGPT1, APOA2, MTHFR, APOC3, IGF1, SELP, FABP3, IL6, TNNT1, TNNT2, REN, CD68*
Cerebrovascular disease	0.0015946	0.04946049	*NTRK2, PRKCH, ACE, ANGPT1, CYP4F2, F11, KLK4, MTHFR, CXCR4, ADM, IGF1, FOS, MPO, RHOA, ICAM1, GFAP, SELP, IL6, TNNT1, TNNT2, REN, TNNI3, CD68, NES*
Cardiomyopathy	1.33 × 10^−5^	0.001700831	*TNNC1, LAMA4, PSEN1, TBX5, MTO1, JPH2, BAG3, GJA5, MYL2, TNNT2, DSG2, TNNI3, CTNNA3, FPGT-TNNI3K, CRYAB, VCL*
Heart conduction disease	0.000268757	0.013461245	*CAV1, IL5RA, DPT, CXCR4, MTHFR, TBX5, SLC4A4, DKK1, ARHGAP24, SFRP2, NFIA, CLDN14, TRPM7, BMPR1B*
**(B)** **Disease**	**q-value**	***p*-value**	**Genes**
Holt–Oram syndrome	3.30 × 10^−7^	2.16 × 10^−5^	*SALL4, TBX5, NKX2-5, TBX3*
Hypertension	0.00045705	0.006255711	*NR3C1, ICAM1, ANGPT1, IGF1, FOS, IL6, TRPV4*
Diabetes mellitus	0.000426195	0.006255711	*UCP3, HNF1A, FOXP3*
Diabetic retinopathy	3.12 × 10^−5^	0.000720179	*IL6, ANGPT1, IGF1, ICAM1*
Cerebrovascular disease	1.25 × 10^−6^	6.14 × 10^−5^	*IL6, ANGPT1, TNNT2, CXCR4, FOS, IGF1, NES, GFAP, ICAM1*
Congenital heart disease	1.14 × 10^−5^	0.000405807	*MEF2C, NKX2-5, TBX5, LEFTY2, NODAL*
Heart conduction disease	0.000629384	0.007274938	*CXCR4, TBX5, DKK1, NFIA*

## Data Availability

Data are contained within the article and Supplementary Materials.

## Supplementary Materials

The following supporting information can be downloaded at: https://www.mdpi.com/article/10.3390/life14040493/s1, Supplementary Table S1. Python analysis results of 3044 hits from Signal Search Analysis Server resulted in 40 representative sequences. Supplementary Table S2. Transcription factors interactions according to the STRING database. The table shows direct interactions between non-members of the KLF family and their target genes. Supplementary Figure S1 CACCC-box binding partners (Non-KLFs). Supplementary Figure S2. KLF and CACCC-box gene interactions: (Left) Hypertrophic cardiomyopathy, (Right) Diabetic cardiomyopathy.
