# Supplementary material for: Is the Cis-Element CACCC-Box a Master Regulatory Element during Cardiovascular Disease? A Bioinformatics Approach from the Perspective of the Krüppel-like Family of Transcription Factors
Source: Life (Basel). 2024 Apr 11;14(4):493. doi: 10.3390/life14040493 (PMC11051458; doi:10.3390/life14040493)
Supplement: Supplementary file 1 [file life-14-00493-s001.zip › life-2940812-supplementary.pdf]

ID	Gene	Motif Alignment
MA1627.1	WT1	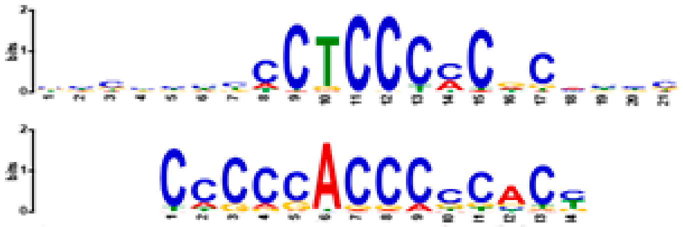
MA1961.1	PATZ1	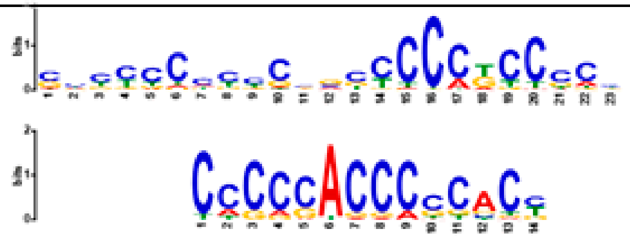
MA0162.4	KMT2A	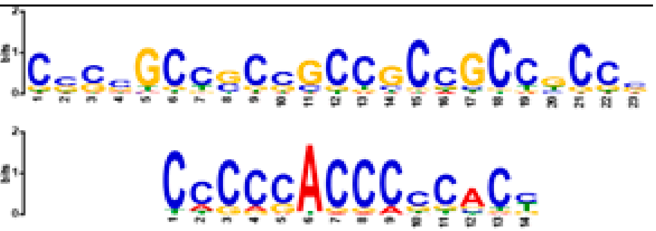
MA0747.1	SP8	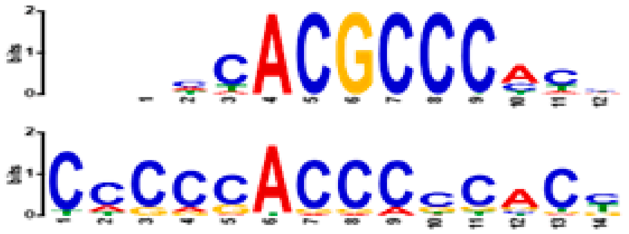
UN0262.1	SALL4	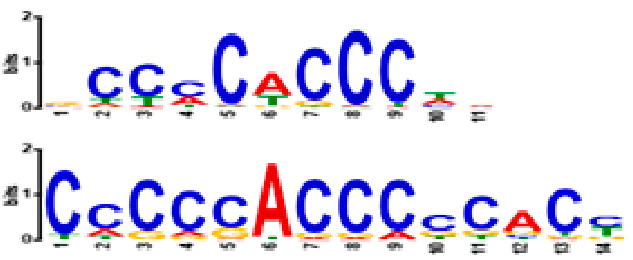
MA1578.1	VEZF1	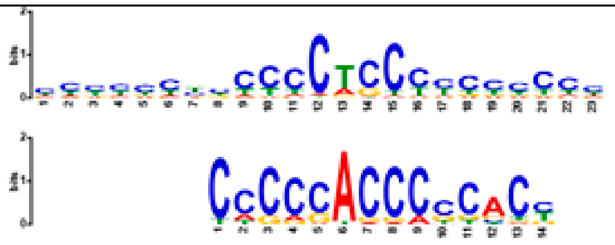

Supplementary Figure S1. CACCC-box binding partners (Non-KLFs).

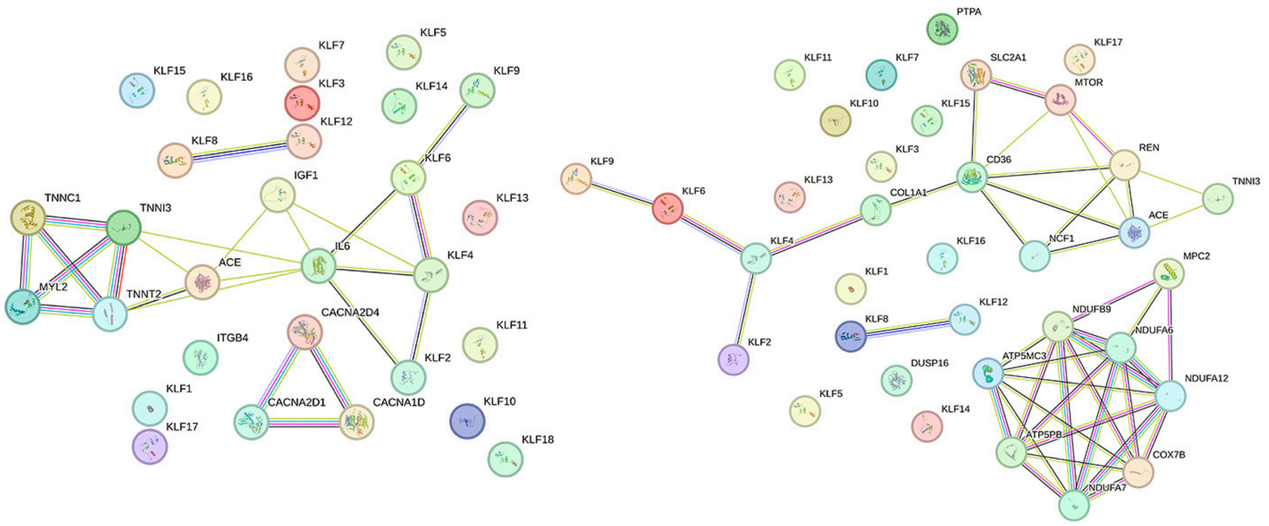

Supplementary Figure S2. KLF and CACCC-box gene interactions: (Left) Hypertrophic cardiomyopathy, (Right) Diabetic cardiomyopathy

Supplemental Table S1. Python analysis results of 3044hits from Signal Search Analysis Server resulted in 40 representative sequences.

Region	Sequence
FP001062	CAGTTAG-GGGTG-CGCTGGAGA-GGGTG-GGGCCCGACTCCGCCA-CACCC-<br>CAACGGTCCTTCCCCCTCCTCACCACTCCCGCCCC-CACCC-<br>CCAATGGATCTGGGACTGCCCCTTTAAGAGTAGTGGCCCCTCCTCCCTTCAGAGGAGGACCTATTAGAGC<br>CTTTGCCCCGGCGTCGGTGACTCAGTGTTCGCGGGAGCGCCGCACCTACACCAGCCAACCCAGATCCCG<br>AGGTCCGACAGCGCCCGGCCCAGATCCCCACGCCTGCCAGGAGCAAGCCGAGAGCCAGCCGGCCGGCG<br>CACTCCGACTCCGAGCAGTCTCTGTCCTTCGACCCGAGCCCCGCGCCCTTTCCGGGACCCCTGCCCCGCG<br>GGCAGCGCTGCCAACCTGCCGGCCATGGAGACCCCGTCCCAGCGGCGCGC-CACCC-<br>GCAGCGGGGCGCAGGCCAGCTCCACTCCGCTG
FP001404	AGCCGAGGTTCTGCTACTCCAGGAGCCGAGGTTCTAACGTGGTCTGCGTCCAGCGCCCC-CACCC-<br>CTACTCC-CACCC-CTACCCC-CACCC-C-CACCC-TTCAACCCGCCCCCTACCCCCAGC-CACCC-<br>CGTGGCCGCGAGCCAAGAGGTCCCTTTAAAGGCGCGAGTCCCGCCCCCACTGCTCATTAGGGCTTTCGAT<br>TGGCGGCTCCTGGGCGGGGCGGGGCCCGGCCTGGGCTCGGGGCTGGCTCGGGGCGGGCTCGGGGCCGGA<br>TCGGCCCCCGCCCCCTTTGTTCGCGCTGCGGCGGGAGGTGGCTGCC-GGGTG-<br>GGAGGTGTGTGCGCGTGTGCCGCGCCGTAGGGAGCGGCGGGTCGCGCCGCCGGCTGTCAGCTAGCTCCG<br>CGCCGCCGCCCGATGGCGCGAGGAGGCGCGCGGGCCCAGCCCCGGGGGGCCGGCCGC-GGGTG-<br>AGCCCC
FP006896	ACCCCCTCGGTAATCCCGTACTCCCTCTAGCGGCGGGCCACTGCAGCGTGGCCACCGCCCC-CACCC-<br>CCATCCCCCATCCCCG-CACCC-CCCTC-CACCC-<br>CCTAGCCCGCGGAGCACGCTGGGATTTGGCGCCCCCCTCCTCGGTGCAACCTATATAAGGCTCACAGTCT<br>GCGCTCCTGGTACACGCGCTTCAACTTCGGTTGGTGTGTGTCGAAGAAACCTGACTGCGCCCTGAGGAGA<br>ACAGCGGAGAAGGTCCACCGAGCCTGGCGAAAGGTCCGCTGAGCGGGCTGTCGTCCGGAGCCACTCCG<br>GGCTGCGGAG-CACCC-<br>AGTGGAGACCGCGCCTGGCTCAGGTGTGGGACCCCATCCTTCCTGTCTTCGCAGAGGAGTCCTCGCGTGG<br>TGAGTATGCGGTGAGGACGTTTTTTTTTAAAGATCACGTTCATTCCCCGTGCTTATCGGG
FP009159	GTCCCAACTGGGAGGGGCCCTGGCAGCATGAAACCCC-CACCC-<br>CCACTCTCTGCGACGGCCCAGATGGAGGGCAGGAGGAACAGCGGTTCAGCCCTGGCTGGGGCTGCAGG<br>GGCTG-GGGTG-<br>TGTTACTCACTGGCACGATGGAGAACTGCATGAAGAGGCAGGTAAGTTCTGTGGCGGCCAGCACGTAGG<br>TAAGCAAGATGACCGAGGACCGGCCTAGAGCGCTCATCCTGCCGGGGGACCGGCCCTGGTCCCTGGGAG<br>CCCGAGCTCCCTGCATCCTGGACAGGCGCAGCAGGGAGACCCAGGAGAGATCCAAGCAGGAGGCTGGC<br>GGAGCAGGGGGCAAAAGTCCAGTTGATCCGGGCAG-GGGTG-CTGAGTCACTGGGTCCCAGGCAG-<br>CACCC-GCCTGGCCTTGCCTGCAAGAGAGGACAGCACTGCAGGACCCCCTCCCCAGTGGAGCT
FP009252	GGTGAGTGTCAGGCCGGCGGCGGCCAGCGACGCGATCCCGCG-CACCC-CGTCGGGGGCCGTTAG-<br>CACCC-<br>TCGCGGGCGCTGCGCCCCTGCCGCCGCCCCGGGGGCAACCCGGCCGCCGAAGAACCCTGCCCGACGTGC<br>CCGCTGCCGGCCTTAAAGGGCCCGTGACCGCTCCCTGCCCC-CACCC-<br>GGGCCGTGGGGAGGCTGCGCCCCTTAAAGGCGCCGCGCCCGTCCGCCCCCATCCTCCTCGCGGGGGCTG<br>CCGCTGCGGGCTAGGGCAGGCAGAGGTGCTGCAGGAGCCCTTAAAGGGGCCGGGAAAGTGTTCGCTGG<br>AGTACCCACCGCCCTTCCGCGGGCATCGCTACCTGGCGGGGGGAGGGGGCAGCTAGCTGCCTCTTAAAG<br>GGATAGCTCTGCCCTTGGGGGCAGTGACTCGGCCGAAGAGGACTCCTAGATGTGTCCCCGGG
FP010074	GGCTGGGACTGGGGTCCCAGGAGGCCCTACCCC-CACCC-TGTGAGAGGTAG-GGGTG-<br>TGGGGAAGGCAGCAGGGGAGGATGGCAATAAGAGGGAGGCTGGGATGAGATCTCAAGCTCTGGGGTCG

	GTACTTCAGAGGGAAAGGAAGAGATTATAAAGGAAGGAAAGAGTAGGGAAGAGAAGAAAATGAGAAT<br>TCCTATGTAGCAGGCTCTGTGCTGGGCACTGCACCTGCAGTCCGTTTTTTTTGTTGTTGTTGTTAGAGTCTT<br>GCTCGGTCGTCCAGATGGAGTACAGTGGCACCATCTCCACTCACTGCAGCCTAGACCTCCCGGGTTCGAG<br>AGATTCTCGTGCCTCAGCCTCCTGAGTAGCTGGAATCACAGGCATGCACCACCACGCCCAGCCTGTCATT<br>GATTTTTACCAAAATTTTCCACCGTTGGAAGGGACATTAGGCTTGGCGA
FP017185	AGACGGGTTTTAACCATGTTGGCGAGGATGGTCTCGATCTCTTGGCCACATGATC-CACCC-<br>GCCTCCGCCCCCCAAAGTGCTGGGATTACAGGCGTGAGCCACCGCGCCCGGTCATGGAGTGAGTTTTGA<br>AGGCTGACTTTATTTGAGGTATTTGAGTCCTGGTGGGGGGGAACCCACACACAGAGAGGAGGGATTCCA<br>AAGTCGTTAATGGGGACCTGGGAAGGAGCATAGGACAGGGCAAGGCGGGATAAGGAGGGGCACCACA<br>GCCCTTAAGGCACGAGGGAACCTCACTGCGCATGCTCCTTTGGTGCCCACCTCAGTGCGCATGTTCACTG<br>GGCGTCTTCCCATCGGCCCCTTCGCCAGTGTGGGGAACGCGGCGGAGCTGTGAGCCGGCGACTCGGGTC<br>CCTGAGGTCTGGATTCTTTCTCCGCTACTGAGACACGGCGGGTAGGT
FP013339	GCCCTTCCTGCCTTCTTGGCCGGGAGGAAGTAGTGCAGCCCCAAAATCAGAGGCC-CACCC-<br>AGCGCCCCAGAGGGCCTGGTCAGAGGTCCTCCCTGCTCCCCAGGAGGGCAGGCGCCCCAGATCCTCTCC<br>CCTCCTTCCTCGCTGGCCGCAGATAAGCCCCAGGGCCCCAGCCTGGCACAGACTGTGGGCACCAGTGGC<br>TTCTGTGCAAGGACTGAAGT-CACCC-<br>AGCAGGCTGCCCTTCCACAGGCGAGATCACCAGGTTCCTGTGGCCCAAAGAGGTGGAGCTGCTGCTGAA<br>AACCTGGCTACCCGGGGA-GGGTG-<br>CTGTGCAAAACCATGTCCTGGTATGGGGCAGGGGACAGAGCCGGGGAGGCGGCTGTGGCCCCACAGAA<br>AGAGCCTCTTGCTCTGCCCAGGATATCAGACTGTCTTTCCCCAGGCACTGCTACGATGGAGAGCCTAC
FP016445	GGCCCAGCTCAGGGCACCGA-GGGTG-GCCGGGTCTGCTGTCAGGTGAGGAGCT-GGGTG-<br>CCTCTGCCGGGGCCCTGGCCCAAGGAGAGTGTCACGGGGAAGGCTGCAGCATGGCC-CACCC-<br>AGGACTTCTGGGGATGAGTCTTGTTTTTCGTTTTTATAGAAACACAGATCTTTGTCCTAACCACAAGGTTC<br>CCCTTTGGTTTATGTGGTTTCCTCTGGTTCCTGCCACCACCACCTCCCCAGGCCGGGGCCTTCCCATCCCTG<br>CCCAGTGGGGAGTGGGAGGAAGAGCCTGGGACAGTGGCCATACGTCCCGGGCTGATTTCTGGATTGGGG<br>GTTCTGGGGACCAGGGAGGTGCAGGTGTCTGCAGTGTCAGAGGAGCCGCTGGGCTCGCCCAGGTGTTGC<br>CTGAAGCCCTCCGTGGCCTCTTGCCCCTTGGACTCCTGACAGGAGGGACACC
FP000345	CGTTTT-CACCC-<br>TGGAAAGGAGTTTGGGTTTTCAAGTGTGACGAGGCGCTATTAAAGAGTTTTAAACATGGAAATTACTCGA<br>TCTGATGTTTGTGTAGTGTCCTGTTTGTTAGTCATCTTTTCCAAACTATAATACATTTTTTTTTCCTAAAAA<br>TGGTCGTTAAAGCAGCCTAGGACTTAGGTCAATTGCGGTGGTCACTTGACCGCCTGTGAGGAGGCGGCGC<br>GTG-GGGTG-GATCGGACTGGGCGTGGCGGGGGCTCAGGAAGGA-GGGTG-<br>GCCCTACCCCAGCGGGCTCGGCTCGGGGCCTCCGCGGCAGTTCGGGGTCCTT-CACCC-<br>GCCGGCTCCAGGTAGGCTACTCCTCAGGTAAGCCCCGCCGCCAGCCGCGACGTCGTCGCAGACAGGCAC<br>CGCCCCCACTCGTGCGGCGCGAGTAGTCCTGCCCCTCCGCGTTGTCTCCGGCCG
FP010429	CTGCAGGAGAGTGGCTTTTATTAAAGCTCTTTTGCTTCTTAGAGAATGTTATCACCACTTAACATCCTCTG<br>CCTCCAATTTAGTGCATTCTTTAAGTCCAGAAA-CACCC-<br>AGAGTAACTGGCTTTTCTTGTTCTCCCTGTTCTGGGAAGTATGAAGGACTGCCCGGAAAAGAGTACAGGG<br>AGGGGGGGATTCTTGATCCATGCACAGCGATGTGAGCTGAGGTGCAGGCACCAGACCTAGGAATTCCTA<br>GAAAAATAGTCAGGAAGCATTTAGACACATCAAATGTTAAACGAGTCCTGATTATGATGATAATGATGA<br>TGATTTTGGTGGTTGCAATAGCAAAGCCTTAAGTATGAAGGAGACTTGCCAGCTGGAAATACAGGTCGGT<br>AGCAGCATTAAGTATCTAGAGGAGCTGCAAATATTATTTTTTTGTGTGCTCATTTTATTTCTTT
FP009059	ATGCAGATTTCTCTGAGCTCTTTAGCGCCCCGAGAAGGGGGAGAAAAACAGGATATTTCAGGCAAACAA<br>ATGAAAGAAGTGCTGCCCTGAAAGG-GGGTG-GTGGTGGGGAG-CACCC-<br>CCAAGCTGCTCGCAAGTTCTGATTGGACGCAAGCATTAAACCGGGAGGGCTTTGTGGTCCTGGGTCAGTG<br>TGTGTTTTTTGAGATTTCAATTTGTTGAGGAATTTCCCCCTAGCCTTGACCCCTTGACAGCTCCCGCTCCTA<br>CTCAGTGCTGGGGAGAAGTAGGGAGGCCTTAAGCGAAGAGATGGGTCTGCACTTTGGAGGAGCCGGAC

	ACTGTTGACTTTCCTGATGTGAAATCTACCCAGGAACAAAACACCAGGTGATCCCAGTGGCCGAGCCAG<br>CAGTGGACAGAT-CACCC-CTTAGGTGGAAGCCCAAAGCGGAGGGCACTGTATTGGTCT
FP005350	CTCAGAGCTGAGGTGGGAGATCTGCTCTTCCAGCCCGCCTCTCCTCCTCCAGCTCCCCAGCTCCACTCAA<br>ATTCAGCTCCCCTCCCCCCTGCC-CACCC-<br>CCGCCACTCGGTGACTAAGAACTGCTGCAAGCAGGCAAACCTCCGAGCAGTTTTTAAGAGGCTGGAAGG<br>AGACATAAGAGACTTCAGCTGCTGCATTCCAAGCCCTGGGTCTACCTTGGAGACAGGACAGCACAGACT<br>TACTCTCCACAAAGGGACTTCTGGGTCATGGGGCCCAAGACACTCCCACAGCTCGCTGGGAAATGGCAA<br>GTGCTGTGCATGTTGTCCTTGTGCTGCTGGGGCT-GGGTG-<br>TCTGGGCAGCTTCGTTACTCAGTGGTGGAGGAGTCTGAGCCGGGGACGCTGGTGGGGAATGTTGCTCAGG<br>ATCTGGGCTTAAAGATGACAGATCTGTTGAGCCGGCG
FP008185	GATTTGAGGCATTGTCATCCCCCG-CACCC-<br>CTCCCCACGTGGCCTTCTGGCACGAGCCGCTGGCTCGCCGCCTCATTTACATCAGAAAGC-GGGTG-<br>CCGGCCAATAGGCGCGCAGCCTCGCGCCAGCTGGCGGCGCCG-CACCC-<br>ACCAGCCTATATAAGGGCGCGCAGCGTAGTAGGGGCGCACTCGCTGGTGGTGGGCGCGCCGTGCTGAGC<br>TCTGGCTGTCAGTGTGTTCGCCCGCGTCCCCTCCGCGCTCTCCGCTTGTGGATAACTAGCTGCTGGTTGAT<br>CGCACTATGACTCTGGAAGAAGTCCGCGGCCAGGACACAGTTCCGGAAAGCACAGCCAGGTGGGTTTCA<br>GGGCGCTGAGAAAGCCGCTGGTCGGTCGGCGACCGTCAGGGTTTTCCA-GGGTG-<br>GGAATCCGCGGGGTAGGAGGGGAGCGCGGGGGTCCGGGCGCCAGATCC
FP009599	CCACAGGACCTGGGCACTGGACCTTATGATGTGCTTCTCTGGAGTGAAGACTGGCTGTCACCTCAGCCCC<br>CTCTCCTTTCTTCTGCTGCCATATCATTCTTCACATGCCTACCAGCCCCCTGGAATCTGCCCCTG-CACCC-<br>TCATCCCTTATATATTTTGCAGAGAGCGTTTAGAGGACTGGAAGTGTGGCATGTAATGGTATCTCTGTTTC-<br>CACCC-<br>CGCAGCCCACCTTGGGACACCTTGACTCCAAGCCCAGCAGTAAGTCCAACATGATTCGGGGCCGCAACT<br>CAGCCACCTCTGCTGATGAGCAGCCCCACATTGGAAACTACCGGCTCCTCAAGACCATTGGCAAGGGTA<br>ATTTTGCCAAGGTGAAGTTGGCCCGACACATCCTGACTGGGAAAGAGGTGAGCACTGGGACTGGGGACA<br>TGGCAGCACCTCCCAGCCCTGTTGACAC
FP003211	TGGACTGTTACAATATTCACTGGCCTATCAGAAGAGGTCAGTTAAATATT-CACCC-<br>AGGCCCTGGGGGCTCTCTTACAGCTGTTCTGGCAGATATTGAAGTAATATGGTCTCATGCGATACAAAAA<br>TACTTGGAAATCCCACTGAAAGATTTAAAGGTGAGCTAAAATCCTCAGTTATTTGGATTGTTTTTAAGATT<br>CTTGGTCAATGAAAGTACACGTTACCGAGTTGTTTGTAATATAGTGTCCTTCTTGAAACATTCTTGACCTT<br>CA-GGGTG-<br>AGGATTCTTGGTGGCATGAGGATGTCTTTTCTTCTTCACTTAATTCCCTTCTGGTGCTTTTTTTCTTTCTCTGT<br>TTCTTTGGTGTCTCTATTACTCGAATCAGCAAGCTTGCTCATTTTTGTCTTCTCTTTGGACCTTACTGACACA<br>TCAGTTGTCTTATTTTTGATTTTACAAGTT
FP003847	CTTTCTTAAAGGAGGAGCTTTCTCCTCTCACCTCAAGGACTGTTATTTATTTGTCTGCGCTACTCTTTCGAC<br>GTTTCCATTCTCTTGGTCCTCAAGATGTGAAT-CACCC-<br>ATTTTTTCCGTAGGGGGGAAGACGGCGTATCTCTGGCCCTTTAAATAGGGGCGTGGTGAAGAGGCGCGG<br>ACCGCCCAGGAGGGGGAGCAGTGCGAGGAGGAAAGCGGAAGCGCTGCCCGCAGTGGTAGGTAGTGCGC<br>GTGCGTGAGGCTAGAGCAGT-GGGTG-<br>CGGTCACGTGGGCCGGCGGTCCACGGCCGGAATGGCAGCAAGCTCAGGTAACGAGTTTCCAGAGAGCG<br>GGCCCGTGGAAGGAGACAGGATTGAGGGGCCGTATTCAGGGTAGGAGGAGAGCGCGCGACAGGTCTCT<br>GAGCCTCGGCGTCTCCTCCCCGCGCGACAGCGGCGGGACCTCGG
FP014009	CCGGGTTTCCTGGGCTACTACGATGGCGATGAGTTTCGAGTGGCCGTGGCAGTATCGCTTCC-CACCC-<br>TTCTTTACGTGAGGCTCAGACCCCAAGAAGCACCGCTGTGCCTCCCTCCCCTCCCCCGGACCCTGGGCTC<br>AGCCCTGATGCTTCATATGGGGAGGGGGAGAAAAGACCCGTCGGCCAA-CACCC-<br>TCAGTCCCTTACTTTCTTCCTGAAATGCGTCCCGGGCAAGTGCTGCCTCCCGCCCTCTAATTCTGCGCCCC<br>ACTCCCTT-CACCC-

	GGCAGGTTACAACCGAATGTGGACACTCGGCAGAAGCAGCTGGCCGCCTGGTGCTCGCTGGTCCTGTCCT<br>TCTGCCGCCTGCACAAACAGTCCAGCATGACGGTGATGGAAGCTCAGGAGAGCCCGCTCTTCAACAACG<br>TCAAGCTACAGCGTATCCTCCCTCAGGCTCTTCCACAG
FP007067	ATGGGGAGTGTAAAGAGGCACTAGCAAAGTCCGAGATGAATGTGAATATGAAGTATCAGCTTCCCAACT<br>TCACCGCGGAAA-CACCC-<br>ATCCAGAATGTCATTCTACATGAGCATCACATTTTCCTTGGTGCCACTAACTACATTTATGTTTTAAATGA<br>GGAAGACCTTCAGAAGGTTGCTGAGTACAAGACTGGGCCTGTGCTGGAA-CACCC-<br>AGATTGTTTCCCATGTCAGGACTGCAGCAGCAAAGCCAATTTATCAGGAGGTGTTTGGAAAGATAACATC<br>AACATGGCTCTAGTTGTCGACACCTACTATGATGATCAACTCATTAGCTGTGGCAGCGTCAACAGAGGGA<br>CCTGCCAGCGACATGTCTTTCCCCACAATCATACTGCTGACATACAGTCGGAGGTTCACTGCATATTCTCC<br>CCACAGATAGAAGAGCCCAGCCAGTGTCC
FP002937	GTTCAGTTCTCTACAGATTTGCTCTTGGGTTTATTATCTGCCCCTC-CACCC-CTGA-GGGTG-<br>TCAGTGCTGTGAGGACGCTGGGAACCTGTCTTGCTGGCTGATAAACCCCTGGTTCCTAGAGGGGCT-<br>GGGTG-<br>CAGAGGAGGCGTTCCTTTAGAAATGGGGCAGGCTTGGGGACCACTGCTGTGGTAACGAGTCCTGCTGTCC<br>CTCTGTCACTGCCCTATGGGGCTGCGCTTTAGGCTGGGAGTCGGAGAAGGAGCTCTTGCCTGTGACTTTG<br>GGGAGCACGCTGTCTATAAGAGTCAGGTACGGCCACTGGGTCACAAGTGGGGCTACAGGCACTGGGGA<br>CTTGGGCCA-GGGTG-<br>GAGGAGGGAGGAGGCATGAGGACTGGTGAGAAGGTGGCATTTGAGTGGCTTGTGCAAGATGAGAGGGG<br>AGAAGAGCCCTTGGGGGAGTTCAGGCCTAA
FP013104	CCGT-CACCC-CCCATCATACCTCC-CACCC-CCTCCGGGAAGGGAAATTACATTTCCCCATTTTAGCAAG-<br>GGGTG-<br>GCGAGAGGGAAGAATGAGTGAACACAAAGCTGAAAGTACAAGCAGGACGGAAAGTGAAACCGGGCAC<br>AGCCCCCAGTATAAGACCCCCCCTACCCAGGAGATGGCTGACCAAAGAGGCTGGGCCCCGCCATGGGC<br>CAGACGGCAGGCGACCTTGGCTGGCGTGAGTTTGAAGCCAG-GGGTG-<br>GGGGGCTTGGTTGAGTTTGGGCAGAGAATGGACGAGCTTGGCTGGTGCAGATGGGGGGTCGAGAGCTGA<br>GAGCAGCAACTTGGCCAAGACTGAGGATTCGGCTGGCTGCTGCATGGGAAGGGAGGCTGGCATGACT-<br>GGGTG-GGGGCCTGGGGCAGTG-GGGTG-GTGGTCTGACCCAGGGCTGGGCAGAGGATGAGAAGG
FP000529	GGACTGGCGGAGGCCGCGAGGGAGGGAGCACGAGCGAGGAGGCACGCGCCCGCCGGTCCG-CACCC-<br>GGCCCGCCGCAGCCGCCGCCGCCGTCGCCGTCGCCGCAGCAGCCATGGCCGAGCGCCGCGCCTTCGCCC<br>AGAAGATCAGCAGGTAAATATCCGGCGTGGGGCGCGCCGCCGCCGCCGCCCGAGAAAGAGCCGCGGAG<br>TCCTCCCAGGCCTGGCCTAGGAGGC-GGGTG-<br>GGTGTGGCGGGGCCGGCACCGGGATCCCGCGGCCTGGCTCGCGGCGGGGCGGAAGGGGTTCG-GGGTG-<br>GCTGAGGGGGTAGCGGAGGCCTCGCGCCGCCCTCGAACCCCGGCCTTCAGCCCGCGCCCGCCGCTGAGC<br>CTCGGCAGGGAGCGGAGAGGGCCGCGGACTCCAGCGCGCCCGCCTCCTCCTGCTCCTCGGCGGCCCGGG<br>CCTGGACTTCTGCG
FP004135	GCGGGTCTCCATCCGTGGCGCCTCGCCAGCTGCAGGACGACGCGGGCTCTTGAGGGGCAGAGCAGCGCA<br>TCATGCAGGTGAGGGTCGGGACCTGCCGAGC-CACCC-TGGAA-CACCC-<br>AGTGGGTCTCCAGCTGGGCCCGAAGCCAAGGGACACAAATGGGGAGGGCGGACACAGGAGGTAGGCTC<br>TAACATTTGTTCTTGGAAAGTGCTAAATTTTGCGTGCTTCTCTGTCTAGTTTTGAGAAAAATCCTCTAGGA<br>GAACGCCATTTTTTCAGCCTTTAAGACTTGTGTCTGTCGCTGGAGAAAACGTTAAAAGAGTTGTTACGTAT<br>TGTGTTTCACACCAAAATCATAGAAATCTTACTGCTGAATACTATTTTTGACTTAATAGAGCGGCAACGTT<br>AGCCCAAGTGTAATTCAATTTGGTTGTCCTCCTCCACTACCTTAAAAAATAAGAA
FP017366	ATTGGTCTCTGTTTTTGTGAATCCCTGTAGGTAGATCTGTCCCCAGGTCTGTGGAACTGTCAGTTGTGAAGT<br>TTTGTAAAATGGT-CACCC-<br>AACTTAAAACTAGGAAATTACGAAGAAGAGAAAATTGCCCTGTATCTGTTAAGGTTGGTGTAAAGGTAC<br>AGGGACAGGGGGGCAACTTCGACTAGAGAATTCCATCTCTTCTGTTTTGTCATTTTACCAATTATTCCCAT

	CTGCCC-CACCC-<br>CCTGCTGCTGCTGATTTAGTTGGGAGTCTTGGTAAGTTGTGGGGGTACAGAATGCTAAGCTGCCTTTCCCA<br>CTCTCCACTGCTTGTCCTGCTCTCACGCTTGAACCTAGTCTAATCTGAACGAGGGACATTCTAGGGCAATC<br>ACTGAAGAGGAAGATGGTGGGCAATAGGAAGGAGGAGGAGGAGTAGCCCTGAAACAAAGCAGTGGGG<br>G
FP003318	GTTCCCCCATCCCAG-CACCC-<br>TCCCTCGCCACCTCGGCCGCGTCCAGAAGCGTCTCATTCAGATAGAGGTACCCAGCCGGGCCCCAGGTCT<br>CACCTGGGTCAGCATTGTCCCGCTGAGAGCTTTCAAACAACAGGTGTACGCGAATCAGTATCAAAAAGC<br>CTCCAGGGAACTGAAAAAAACAGTCTGACTTCCGGGATTCGCGGGCATCCACTTTCATTCTCACGTTTCC<br>TCTTCAATTTCTCGCGAGATACTGATTTGCTCGCGCTATAGAATTCTTGTCGAAGAGGCTTAGCTCCCGCG<br>GGAGATTTCGTGCGCAGTTACTTCCGGTAGCTGGTAAAGGCTGATACTTCCCAGGACGCGGAGGTAACG<br>GGCCAGGGCCAAAGCGACTTTCGCTACTTGGATTGGTCGGCGTAGCTTTGGGCGGCCGGACCTTAGAAA<br>GTCACACATCTGC
FP001165	GTCACAAGGTTCC-CACCC-<br>ACTGGAGTCCCTCCCCGTGTGCCACCAACCAGCACTTTTGTCATGCTGGGAAAACCTCC-CACCC-<br>AGGCCTTTGCCCTGAGTCCAGGGTCTGTGCCCTAACCCCACAGTCACTGAGAGCAACTGGAGGCCACAT<br>AAAACAGACATCTCTTGTTTTCCAGATACTACGGGTCATAATCCCTAACTCCAATCACTGGCAACTCCTG<br>AGATCAGAGGAAAACCAGCAACAGCGTGGGAGTTTGGGGAGAGGCATTCCATACCAGATTCTGTGGCCT<br>GCAGGTGACATGCTGCCTAAGAGAAGCAGGTAGGAAATCTGTTGGGGACCAAAGACTAAATGTAATAA<br>AATACAGATAATGCTGGGGGCTAGGCATAGTGGCTCACGTGTGTCATTCCAGCACTTTGGGAGGCCAAG<br>GTGGGGGGATCGCTTGAGCCCAGG
FP001272	TTTTTAAAAAGAAACTCCAAACAGACTTCTGGAAGGTTATTTCTAAGAATCTTGCTGGCAGCGTGAAGG<br>CAACCCCCCTGTGCACAGCCC-CACCC-<br>AGCCTCACGTGGCCACCTCTGTCTTCCCCCATGAAGGGCTGGCTCCCCAGTATATATAAACCTCTCTGGA<br>GCTCGGGCATGAGCCAGCAAGGC-CACCC-<br>ATCCAGGCACCTCTCAGCACAGCAGAGCTTTCCAGAGGAAGCCTCACCAAGCCTCTGCAATGAGGTTCTT<br>CTGTGCACGTTGCTGCAGCTTTGGGCCTGAGATGCCAGCTGTCCAGCTGCTGCTTCTGGCCTGCCTGGTGT<br>GGGATGTGGGGGCCAGGACAGCTCAGCTCAGGAAGGCCAATGACCAGAGTGGCCGATGCCAGTATACC<br>TTCAGTGTGGCCAGTCCCAATGAATCCAGCTGCCCAGAGCAGAGCCAG
FP013976	CTCGCCCTTCTTGCCCTGCTTTTCTTACTGTGCTCTGTTCCGTCACCACTAGCTCCCCTCCCGCGCGGCTCT<br>GAGTCCCGCTTGCCACTAGTTGCTTCTCGGCAGCCCC-CACCC-<br>CTACGCGTGGTCCGGCCCGGCGGCTGGCGCAGGTTATAAAGGTCCCGCCGCAGCAGCGGACGGCAGACT<br>CGAGATGCGCCCGAAGAGGTTGGGCCGGTGCTGCGCGGGGAGCCGGCTCGGACCCGGGGACCCAGCAG<br>CACTCACCTGTGCACCTTCGCCCTCAGCCAGTCCCGCTCCGGAGCCCTCTGCGCAGCCGCAGGCACGGGG<br>CACTGGACAGAGAGTAGGATCCCGAGCCACCTCTGGATCCCAGTTCCTCTCGGAAGCCCGCACCGGAGC<br>TCGCCCGGCCTCGGAGGCTGGAGCCAAGGCAGGAGCCCGGCGCCCGTCCGCATTCTCGGCC
FP017441	GCTCTCCAGCAGCCCAGCCCTTTCCCAGGGCTGGGGCAGGAGATTGCTACATGTAGGCTTATCTGGGGAA<br>AAACCAGAGCCTCACTTTAGTCCCTTCCGGTAATTGACACTACTGGA-CACCC-<br>AGGAGGGGGAGGAGAGAGCTTCTCTTCATAAATGTTCC-CACCC-<br>CTGGGCAAGGTGGCTCACTCTGGCAGGTAGGAACAGGGGAGAGTGCACCTGCTACCAGTCAAGCTCAGC<br>CAGACTGCAAGAGGAGGCGAGGCGGAGCCAGCCGAGGGAGTGAACCATGGACAAGTTGAAATGCCCG<br>AGTTTCTTCAAGTGCAGGGAGAAGGAGGTAGGGGTCTGGGAGCTGCGGGAGGTGTGGAGGACCTGAGA<br>GTGGAAAACTTAAGGGGGGTTGCTAGTCTCAGTTTTTGCTTTCTGTGGCTGTTCCTTGTGCTCCACATTTCT<br>GTTCAACTAT
FP003053	AGGATTAGAAAGTGGAGCAGCAACCGGAGGCGTCTCTGGGCCGCCCCCCCAACCCCCC-CACCC-<br>CGCCTCGCCCACTC-CACCC-<br>TCACACTGGCGTTGCCACAGTCCCTCCATTCCAGAGGGAATTCCAGAGCGGAATACGGATTCCGATTTA

	AAATAAGCGCTTCTAAATCTTTCTCCTTGCATCTAAGAC-GGGTG-<br>GGGGAAAGAAAGGGAGAGGAGTTGAGGTCCCAAGGTTCAAGGCCTGGTGATCTCCAGGTCCCAGGCTG<br>GGCGGCCTCGGTGCACGGAGAGGGCGAGGGGCGTGTTGTGGAGCCTTGGCAAGGCGCTGCTCTTCCTCG<br>CGCCTCGGTTTGCGGTCTGTGAAATGGGGTTGGCAGTGCCTTTGCAGGGACGCGGCGAACTCAAGCACCA<br>GCAAGCGGACTCGAACTCAAAGGGTTTCA-GGGTG-TAGACGACCAGAGC
FP002378	GTGAGCGACGTCCATTT-CACCC-ACCTCGCCGCGACC-CACCC-<br>TGGGATATGTAGTGCGTGACTTCCGCAGTGCACGCTGCGCCTGCCGGGCATTTTTTCCTGGTTTGGGGGCA<br>AGGTCTCATGGGAAAGGTCATGTCTCTCGAAGAAAGGTTATAAACCCTGAGATATGAGGGTTGGGCGAG<br>ACATCCGAGCCTGTTTCGTTCCGTGTTGGGACCAGGAATAACCCTGACTTCTGAGCTTTCATAACCCCAG<br>GATCCTCCAGAAAATTTGCGGCGCGCTGAGGGAAAACCTTGCTGGCAAGTCGGGACTGTGTGGCAGCGG<br>CTATTTTTCTTTTCC-CACCC-<br>AGCACGTTTTCGGGCTGGGGACATAGGGTTCAGTTGAGATACGGAGGCTTCCTTCGGGATATTGGGTTCT<br>CTGAAGTGCGGATGGACTAGGGCAGGGCTGAGACCCCATTC
FP010292	CAGGTTCCTTCCTCAGGAAAAGCCAAGGTCAGGCGCAACTTTGCCAAATCCCTGCCCCTCCTTCAGGCTG<br>GAGACAC-CACCC-<br>CCAAACTCACCATGCGGGGAGGGAAGAAGTCTGGTAGGGACTCTTATTGCAACAGGGCCCCAGCCTTAT<br>AAAAAGCCCTGGTAACCCGGCCATTACACATCCTGTCTCTGTGTGTGGAGCTGGCTGGAATCTCTCAGCC<br>TCACCTGCCAGACAA-CACCC-CCTCCTTCCT-CACCC-<br>TGTCTCCTGCATTCTCCTGAAACCTTCATCCACACAATGCCTCCCAACCTCACTGGCTACTACCGCTTTGT<br>CTCGCAGAAGAACATGGAGGACTACCTGCAAGCCCTAAGTAATGGCCTCGCCCTCCCAGACCCCTGTCA<br>CTCCCTTTCTCCCCTCTTCCTTCCTGCTTCTTGGTCCCATGGAGAAGAGGGAATG
FP001502	CTGTGTGGGGTAGGGCAGGGTCCACACTCCTTCCCATCCATTTTAGAGGAGGAAGCTGGAGTCTGGGAA<br>GGGATGGGATTTTCCCAGGG-CACCC-<br>TGTGAGTCACATGCCACTTGAGACAAGGGTCTAGAGCTCCAGCATTTTCCAAGCTACAAATGTATCTGCT<br>GCTCCAAGTGTCCTAGATCAGGACCCTAGGTAAAGACTTCTGAAAAATACTTGAGTCTAGAAACAGAAA<br>AGAAAAAGGGCTAGTGTGCTCTAGGGGTTTGCACTGTTGTACCCAGTTTGCTTTGCTGTGTCCTAGGAAA<br>GGTCCTTTCTGGGGAT-CACCC-<br>CATTGGCTGAAGATGAGACCATTCTTCCTCTTGTGTTTTGGTAAGTACCCCTTCCCATCCTTCCAGGCCCTG<br>TTATATGGCAGGGAGGCAACAGCCACAGGGAGAAGGAGAGTTTACCAAAGTGTGG
FP011777	TCTGGGCTGAATAGGTGGTCCACTCTGAGTCATCAGCTGT-GGGTG-<br>ATGATGTGGTCACTGCATGATTCTCACACAAG-CACCC-AGAGGACGTCATCAGGCAGAGGCAGTGG-<br>GGGTG-<br>GGCAGCATTTACAGAAAATCTGTGATGAGACACCACAAAACCAGAGGGGAACATGAAGTCACTGAGCC<br>TGCTCCACCTCTTTCCTCTCCCAAGAGCTAAAAGAGAGCAAGGAGGAAACAACAGCAGCTCCAACCAGG<br>GCAGCCTTCCTGAGAAGATGCAACCAATCCTGCTTCTGCTGGCCTTCCTCCTGCTGCCCAGGGCAGATGC<br>AGGTGAGTGACCGTCTC-CACCC-<br>TCGGGGGCCCAACCCCATCCCACAGGTCTCCTGCCCTTTCTCCACATTCCTGATCCATCTATCTACCAGGA<br>ATGTTCTGAACTCCAGCTCCCATTCTACCAAGACCC
FP010713	CATACGAATCTAGCCCGGGAACCGAGTTGCGGGAGTGCGGTCTGTGCCGTTCCGGCCAGGAGTTTGCCG<br>ACTGCAGACGTCCTGCGAACCGGCAAGATGTGCTCTCTGGGGTTGTTCCCTCCTCCACCGCCTCGGGGTC<br>AAGT-CACCC-<br>TATATGAGCACAATAACGAGCTGGTGACGGGCAGTAGCTATGAGAGCCCGCCCCCCGACTTCCGGGGCC<br>AGGTGGCCAGGGCGAGGAGGGAGGGGACCTACAGGCATGGGGGCGGAGTCGGGGACTACTGGGGAAA<br>GGAGGCAGTGTGACAGGGGCAGGGTCACAGAGTCTGGCGGAGTGGAAACAGCTTGGCCCCGGCAGGAG<br>GCTAGAAGGGCTGGAATCTGTTTTAGGACTGGGATTGGAGTATAACGGGGGAACGGTTAAGGAGCTGTG<br>GAGGAGGCCTAGAGGGAATGGAGAGAATGA

FP015337	AGGTAGGGAGAGAGGAGCTGAGGCCCCAGATCAGCGGCCGCGGGCAAGGTCGCTCAGCGGG-CACCC-<br>GGCCTGGGTATCGGGGCGCGGGTCGGGGGCGGGGCCGGGGCTCAG-GGGTG-<br>GGGCCGGGCCGGGCCGGGCCGGGCGCCTGCGCCATAAAGGCCGCCGCGCGCCCACGCGCCTCGCTTGCT<br>GCGCGCTGCCGGCGCTCCTTCCTCCTCGGCTCGCGTCTCACTCAGTGTACCTTCTAGTCCCGCCATGGCCG<br>CTCT-CACCC-<br>GGGACCCCCAGTTCCAGAAGCTGCAGCAATGGTACCGCGAGCACCGCTCCGAGCTGAACCTGCGCCGCC<br>TCTTCGATGCCAACAAGGACCGCTTCAACCACTTCAGGTGCGGGCGGGCCGGAGGCGGGGGCTGCCACG<br>CGCGGCGCCCGGAACCGGGCACGCGGGCCTGGGGTCTGGAGGCGGGGG
FP000316	GGCCTCCGGGGGAGAAGACTACAGTAAGGGCGAGCAGAGCTGAATGCTTTATGTCGGAATCGACCAAT<br>GGGATGGCGG-CACCC-<br>GGTTGCCAGGGCTACGGCCAAGCGGAGGTAGGGCCTCGGCACTATGGGCGGGGCCTGGGCGGGGTTATA<br>TAAGCCAGCGGCGGGCGGAAGGCGGGGCGTGGGGGTCTGTGGCTGCTGGGCTGGCGGGGCGCAGGCCG<br>CGGGACCCGAGCCCGGGGAAGCGAGAGAGCGGAGGCGCCGAGGATCCGATTCACTCCCTGGGGAGACC<br>TATGGGCCGAAGCCGTGTAAATGCGTTTTAAGGCGAGTGCGGAAAGTGGCCTGGGGAGCCTCGGGGAGC<br>GGACGCCCTCCGCCCTGGTGCTGACCTGCCTCCCTGCCCCTTCTGCCTCCTGTCAGCAGAGGCCTCGGCTC<br>CGCAACTGCCACTCCTCCTCGGG
FP002584	CCCTCCCCTTGGCTGCTCCCC-CACCC-<br>ATTTCCCGCCTTGTCTTTCCTCTCTTCTCTCTCTCCCTCCTCCCTGCGCGAAGCGGAAGTGACGCGAGGCGT<br>AGCGGAAGTTACTGCAGCCGCGGTGTTGTGCTGTGGGGAAGGGAGAAGGATTTGTAAACCCCGGAGCGA<br>GGTTCTGCTTACCCGAGGCCGCTGCTGTGCGGAGACCCCC-GGGTG-<br>AAGCCACCGTCATCATGTCTGACCAGGTACCGGGCTGGGAGCCATCCAACGGCGGTGGCCAGGGCCTTC<br>CCTGCAGGTCTGCCTGCCGCTAGGCTGGGGTCGGGGATGGAGGCGCGGGGGAGGGGAGCGCGGAATGG<br>GGGAGGGGGCGGCCGGTGGGGACTAGGCAGGCGAGGGAGAGGAGCCGACTACGGCCGGAGGTGCCGG<br>GGCCTGCTGCGGGGCTCCCGGAGCCGCCCGCCCCT
FP006713	GGGCAAGCCTGGCAGGGCAGAGGGAGCCCCGGCTCCGAGGTTGCTCTTCG-CACCC-<br>GAGGATCAGTCTTGGCCCCAAAGCGCGACGCACAAATCCACGTGAGTGTTTTCAAATTGAATTTCAATAG<br>GAAAACTTGGGGTAACTGGTGAATTTAAAAAAAAAAAAAACACAGTAAAGAAAAGCGGTAAGGTTGGTA<br>GACCCTGGTGTCGCTCAGGTCCGCCTCTCTTTTCTGAGGACAGTGAGAGAGTTCACTTCTGTCAAGCGTCT<br>GTTGCTCTGCACTGTGCCAGCAGGTGCAGGACCAGGCCGACATGGGACACTTCTGAGCAGCCCCGCTGT<br>CACCAGGAGAGGAGTTCTAGCTCCCAACCATATTTAAATTTATGTAGACCTACATATACCCACGGAAGTC<br>AGCCTTTATAAAGTCGTGTGTAAAGAGTTTTCCTTATATTTGAGCCGG
FP016947	CAGCCAATTAGAGCCCGGCCAGACACCGCGACCCCTCCCCC-CACCC-<br>GCCCCACTCTCGCCCACTGGCGATTGGGTTGCGGGACGCGCGGGGCGGGGCTTCCGGGCGGGGCAGGCT<br>GGCTTCCGGCGGGAGCGGGGCCGCGGATGGCCGTATACAGGGCAGCGCTCGGGGCTTCGCTCGCGGCTG<br>CCCGACTCTTGCCCCTCGGTCGCTGTTCCCCGTCGCCGGCGCCCCGCTCTACGTTGTCGGGCGCCGCCATG<br>GAGCCCGCGCCTCGCTGGCTGGCGGGGCTGCGCTTCGACAACCGCGCCCTGCGCGCCCTGCCCGTGGAG<br>GCGCCGCCGCCCGGTCCCGAGGGCGCCCCGTCCGCGCCGCGGCCCGTGCCCGGGGCCTGCTT-CACCC-<br>GCGTGCAGCC-CACCC-CGCTGCGGCAGCCGCGCCTCGTGGCGCTGTCAGAGCCCGCGCTGG

Supplemental Table S2. Transcription factors interactions according to the STRING database. The table shows direct interactions between non-members of the KLF family and their target genes.

Transcription factor family	Gene	Direct interaction
SP	SP1	COL1A1, NR3C1, TBPL1, TBPL2 MYOD1, HBZ, MDM2, EPAS1, ICAM1, LEF1, RB1, NCOR1, MBNL1, FASN, FOS, HCFC1, PRKACA, DHFR2, HNF1A, MEF2C, IL6, DMPK, MTOR, FHL2, TFDP2, NF1, FDFT1, NCOA1, TFAP2A, ESR1
SP	SP2	FHL2
SP	SP3	SLC20A1, NF1, SUMO1, DYRK1A, ESR1
SP	SP4	NRL, GJA5
SP	SP5	PEX11B
SP	SP6	HHEX, JAZF1, FHL2, CA1, CAMK1D, TCF7L2
*SP	SP7	ACP5, STUB1, COL1A1, SOX9, CD68, LEF1, FGF18, PLOD2, TNFRSF11B, SOST, FOS, FGF3, MEF2C, DMP1, ADAMTS4, NES, DKK1, IFITM5, IGF1, MEPE, CCK, IL6, ACAN, CD44, FGF16, ESR1, DLX5
SP	SP8	PVALB, LEF1, GAD1, BAG3, TBR1, LHX6, DLX5, ENO1, PAX6
SP	SP9	LHX6
WT	WT1	IL2, IFNG, MLH1, FLT3, SOX9, MYOD1, KRT5, THAP1, MDM2, SULF1, PAX8, EPCAM, LEF1, STAR, ATM, WNT9B, RAD21, MN1, TBX5, NKX2-5, GATA2, NF1, NUP214, RBMX, NFIA, CSF3R, AMER1, VIM, PBX1, TNNT2, CD44, FBXW7, PMEL, CITED1, CD8A, MYH11, IDH1, ESR1, PAX6, IGF2, TET2
PATZ	PATZ1	SNRPC, ESR1, CD8A, NR4A3, NCOR1
SALL4	SALL4	TBX3, LEFTY2, ADAMTS18, SOX15, TBR1, EPCAM, PAX8, ALPP, GJA5, KDM2B, EPAS1, TET2, TBX6, PBX1, EED, NKX2-5, TBX5
